# Etiology of hyperglycemia in critically ill children and the impact of organ dysfunction

**DOI:** 10.5935/0103-507X.20180051

**Published:** 2018

**Authors:** Seham Awad El-Sherbini, Huda Marzouk, Riham El-Sayed, Sarah Hosam-ElDin

**Affiliations:** 1 Department of Pediatrics, Cairo University - Cairo, Egypt.; 2 Department of Clinical and Chemical Pathology, Cairo University - Cairo; Egypt.

**Keywords:** Hyperglycemia/etiology, Child, Critical illness, Homeostasis, Insulin resistance

## Abstract

**Objective:**

This study aimed to study the incidence of stress hyperglycemia in critically ill children and to investigate the etiological basis of the hyperglycemia based on homeostasis model assessment.

**Methods:**

This was a prospective cohort study in one of the pediatric intensive care units of Cairo University, including 60 critically ill children and 21 healthy controls. Serum blood glucose, insulin, and C-peptide levels were measured within 24 hours of admission. Homeostasis model assessment was used to assess β-cell function and insulin sensitivity.

**Results:**

Hyperglycemia was estimated in 70% of patients. Blood glucose values ≥ 180mg/dL were associated with a poor outcome. Blood glucose levels were positively correlated with Pediatric Risk for Mortality (PRISM III) score and number of organ dysfunctions (p = 0.019 and p = 0.022, respectively), while insulin levels were negatively correlated with number of organ dysfunctions (r = -0.33, p = 0.01). Homeostasis model assessment revealed that 26 (43.3%) of the critically ill patients had low β-cell function, and 18 (30%) had low insulin sensitivity. Combined pathology was detected in 2 (3.3%) patients only. Low β-cell function was significantly associated with the presence of multi-organ dysfunction; respiratory, cardiovascular, and hematological dysfunctions; and the presence of sepsis.

**Conclusions:**

β-Cell dysfunction appeared to be prevalent in our cohort and was associated with multi-organ dysfunction.

## INTRODUCTION

Critically ill patients often develop endocrine and metabolic changes, particularly disruptions of glucose homeostasis that result in hyperglycemia and hypoglycemia.^(1)^ Stress hyperglycemia commonly occurs in children with critical illnesses.^(2-16)^ Several studies have investigated the adverse outcome of stress hyperglycemia in critically ill children and have shown that it is associated with prolonged duration of stay in the intensive care unit (ICU).^(3,5,6,8,9,15)^ Other studies have demonstrated that stress hyperglycemia is significantly associated with mortality.^(2-14)^

Stress hyperglycemia results from increased gluconeogenesis relative to the clearance of glucose as well as from the development of insulin resistance affecting glucose uptake. These mechanisms are mediated through increased production of counteracting hormones (i.e., epinephrine, norepinephrine, cortisol, glucagon, and growth hormone).^(17-20)^ Furthermore, stress hyperglycemia is associated with pro-inflammatory cytokines, oxidative stress, and therapeutic interventions. Those factors in turn inhibit the secretion of insulin by pancreatic β cells through α-adrenergic receptor stimulation, interfere with insulin receptor signaling and/or insulin-regulated glucose channels, and directly interfere with proper glucose transport and utilization in peripheral cells.^(6,20-30)^

Few research studies have investigated the pathogenesis of hyperglycemia in critically ill patients.^(1,5,31-34)^ The assessment of β-cell function is difficult because of the complexity of the β-cell response to secretory stimuli. Homeostasis model assessment (HOMA) is considered a good method for assessing β-cell function (HOMA-%B).^(35)^

The aim of our study was to assess the incidence and associations of hyperglycemia in critically ill Egyptian children on day 1 of admission and to investigate the possible underlying mechanisms of hyperglycemia in these children through the use of the HOMA model previously used in adults.

## METHODS

In this prospective observational cohort study, 80 patients were screened, and 60 critically ill children were ultimately enrolled. Our cohort was admitted to one of the ICUs of the Cairo University pediatric tertiary care hospital during the period from April 2014 to September 2014. Twenty-one age- and sex-matched healthy children were assigned to the control group.

All critically ill children admitted to the pediatric ICU who were older than 1 month were included in the study. None of the study population had endocrine disorders or inborn errors of metabolism with glucose deregulation or severe hepatic insufficiency. Critically ill children who remained in the pediatric ICU for less than 24 hours were not enrolled in the study.

Sepsis and its grades (sepsis, severe sepsis, and septic shock) were defined according to International Pediatric sepsis consensus conference^(36)^ and Proulx et al.^(37)^ as follows. Sepsis was defined as a systemic inflammatory response syndrome (SIRS) plus infection (any positive culture obtained immediately prior to or during admission to the pediatric ICU and/or clinical evidence of infection). Severe sepsis was the presence of sepsis plus one of the following: acute respiratory distress syndrome; 2 or more other organ dysfunctions; or cardiovascular organ dysfunction, which was defined as hypoperfusion or hypotension responding to isotonic intravenous fluid bolus < 40mL/kg and no need for inotropic support. Septic shock was defined as sepsis plus cardiovascular dysfunction (hypoperfusion or hypotension not responding to isotonic intravenous fluid bolus ≥ 40mL/kg in 1 hour and need for inotropic support).

Organ dysfunction was defined according to the criteria of Goldstein et al.^(36)^

The severity of illness on admission was defined according to the Pediatric Risk for Mortality (PRISM III) score.^(38)^

Previous studies used different cutoff values for stress hyperglycemia.^(3,39)^ We preferred to use the following cutoff values for stress hyperglycemia: blood glucose (BG) levels exceeding 126mg/dL (7mmol/L), and BG levels ≥ 180mg/dL (10mmol/L) to estimate the possible variabilities in incidence and association with outcome. Hypoglycemia was considered when BG levels were below 60mg/dL (3.3mmol/L).^(15)^

β-cell function and insulin sensitivity were determined on the basis of HOMA^(35)^ using blood glucose, insulin, and C-peptide levels. Paired insulin and glucose levels were used to calculate insulin sensitivity (HOMA-%S), while paired C-peptide or insulin levels along with glucose levels were used to calculate β-cell function (HOMA-%B).

Normal HOMA-%B and HOMA-%S values are 100%. Insulin resistance was defined by HOMA-%S being < 50%, and β-cell dysfunction was defined by HOMA-%B being < 50%.^(35)^

The following data were recording for enrolled patients on admission: assessment of organ dysfunction, classification of sepsis grade if sepsis was present, PRISM III score calculation, and calculation of β-cell function and insulin resistance by HOMA. Complete blood count, C-reactive protein levels, serum blood glucose levels, serum insulin levels, serum C-peptide levels, blood urea nitrogen levels, serum creatinine levels, liver function tests, blood coagulation profiles, arterial blood gases, and cultures were all determined on day 1 of admission.

Ongoing assessments included the duration of the pediatric ICU stay, duration of mechanical ventilation if needed, and the outcome.

Patients were followed for their blood glucose levels upon admission (serum BG), and every 2 hours (by glucometer).

Patients with glucose derangements were enrolled after two abnormal readings within the first 24 hours of admission and then blood samples for glucose, insulin and C-peptide were withdrawn.

Blood samples for serum insulin and C-peptide were collected in a sterile plain vacutainer tube. Samples were centrifuged, and serum was stored at -20ºC until the time of analysis. Serum concentrations of C-peptide and insulin were measured using commercially available enzyme-linked immunosorbent assay (ELISA) kits (Monobind Inc., USA). The detection limit for C-peptide was 0.025ng/mL, while that for insulin was 0.75µIU/L.

Informed written consent was obtained from the parents of all patients and controls. The study design conformed to the Revised Helsinki Declaration of Bioethics and was approved by the Ethical Scientific Committee of the Department of Pediatrics, Faculty of Medicine of Cairo University.

### Statistical analysis

Precoded data were entered on the computer using the Microsoft Office Excel software program (2010) for Windows. Data were then transferred to the Statistical Package for Social Science software program, version 21 (SPSS) for statistical analysis. Data were summarized using mean, standard deviation for quantitative variables, and frequency and percentage for qualitative variables. Comparisons between groups were performed using independent sample t-test or one-way ANOVA (if parametric) and the Mann-Whitney test or Kruskal-Wallis test (if nonparametric) for quantitative variables; chi-square or Fisher's exact test were used for qualitative variables. Spearman correlation coefficients were calculated to signify the association between different quantitative variables. P values less than 0.05 were considered statistically significant, and p < 0.01 was considered highly significant.

## RESULTS

This study included 60 critically ill children (32 boys and 28 girls). Their ages ranged from 1.92 months to 11 years (median 1.5 years). The control group included 21 children (15 boys and 6 girls). Their ages ranged from 2.4 months to 12 years (median 3 years).

The demographic, clinical, and laboratory data of the critically ill children are summarized in table 1.

**Table 1:** Demographic, clinical and laboratory data of critically ill children in our cohort

Variables	Total patients n (60)
Age (years)	1.5 (0.16 - 11)
Sex	
Male	32 (53.3)
Female	28 (46.7)
Length of stay (days)	8 (2 - 58)
PRISM III score	7 (1 - 32)
Need for mechanical ventilation	37 (61.7)
Mechanical ventilation duration (days)	6 (1 - 22)
Multi-organ dysfunction	39 (65)
Number of organ dysfunctions	2 (1 - 7)
Respiratory dysfunction	44 (73.3)
Cardiovascular dysfunction	29 (48.3)
Neurology dysfunction	29 (48.3)
Metabolic dysfunction	19 (31.7)
Hematologic dysfunction	13 (21.7)
Hepatic dysfunction	8 (13.3)
Renal dysfunction	3 (5)
Sepsis	32 (53.3)
Sepsis grades	
Sepsis	12 (20.0)
Sever sepsis	5 (8.3)
Septic shock	15 (25.0)
Postoperative	11 (18.3)
Mortality rate	16 (26.7)
Glucose level (mg/dL)	139.5 (27 - 600)
Insulin level (µ IU/L)	5.7 (0.2 - 277)
C-peptide level (ng/mL)	0.8 (0.02 - 10)
β-cell function (%)	49.7 (5 - 240)
Insulin sensitivity (%)	66 (4.8 - 215.2)

Results expressed as median (range) or number (%).

The median [minimum - maximum] BG level of the cases was 139.5mg/dL (27mg/dL - 600mg/dL) compared with controls; 70mg/dL (65mg/dL - 94mg/dL), p < 0.001. The median [minimum - maximum] insulin level of the cases was 5.7µIU/L (0.2µIU/L - 277µIU/L) *versus* 1.5µIU/L (0.2µIU/L - 13.2µIU/L) of the controls, p = 0.003. The median [minimum - maximum] C-peptide level was 0.8ng/mL (0.02ng/mL - 10ng/m) *versus* 0.4ng/mL (0.02ng/mL - 4.8ng/mL) of the controls, p = 0.4. The median [minimum - maximum] beta cell function was 49.7% (5% - 240%) *versus* 135.5% (53.5% - 380%) of the controls, p < 0.001. The median [minimum - maximum] of insulin sensitivity was 66.0% (4.8% - 215.2%) *versus* 108.4% (57.5% - 245%) of the controls, p = 0.04.

Hyperglycemia (BG ≥ 126mg/dL) was present in 42 (70%) critically ill patients; normal BG levels were found in 16 (26.7%) patients; and hypoglycemia (BG < 60mg/dL) was identified in 2 (3.3%) patients.

We stratified critically ill patients with hyperglycemia according to their BG levels into 2 groups: 22 patients (36.7%) with a BG level of 126 to 179mg/dL and 20 patients (33.3%) with a BG level ≥ 180mg/dL. The comparison between study variables and different BG levels is shown in table 2. Patients who had BG ≥ 180mg/dL tended to have the worst grade of sepsis and showed the poorest outcome.

**Table 2:** Comparison between critically ill patients with different BG levels regarding study variables

Variables	Glucose level			
	Normal (n = 16)	126 - 179 (n = 22)	≥ 180 (n= 20)	p value
Length of stay (days)	6.5 (3.0 - 16.0)	7.5 (2.0 - 25.0)	12 (4.0 - 58.0)	0.09
PRISM III score	4.5 (1.0 - 13.0)	6.0 (1.0 - 16.0)	14 (1.0 - 30.0)	0.001
MV duration	4 (1.0 - 10.0)	10 (2.0 - 22.0)	7 (1.0 - 20.0)	0.2
Need for MV	2 (12.5)	13 (59.1)	17 (85)	0.01
Mortality	3 (18.8)	2 (9.1)	10 (50.0)	0.008
Multi-organ dysfunction	8 (50.0)	12 (54.5)	17 (85)	0.049
Number of organ dysfunctions	1.5 (1 - 3)	2 (2 - 22)	3.5 (1 - 7)	0.003
Respiratory dysfunction	8 (50.0)	15 (68.2)	20 (100)	0.002
Cardiovascular dysfunction	6 (37.5)	10 (45.5)	12 (60.0)	0.4
Neurological dysfunction	6 (37.5)	8 (36.4)	13 (65)	0.1
Hematology dysfunction	2 (12.5)	2 (9.1)	8 (40.0)	0.03
Hepatic dysfunction	0	2 (9.1)	5 (25)	0.06
Renal dysfunction	1 (6.3)	0	2 (10)	0.3
Sepsis grade				0.03
Sepsis	5 (83.3)	4 (36.4)	3(21.5)	
Severe sepsis & septic shock	1 (16.7)	7 (64)	11 (78.5)	
Insulin level (µ IU/L)	8.5 (0.4 - 40.0)	4.9 (0.2 - 28.6)	5.2 (0.3 - 277.0)	0.4
C-peptide level (ng/mL)	1.3 (0 - 2.8)	0.6 (0 - 4)	1.1 (0 - 10)	0.1
β-cell function (HOMA-B%)	124.5 (31.1 - 380.0)	37.0 (15.5 - 125.0)	12.9 (5.0 - 72.8)	< 0.001
Insulin sensitivity (HOMA-S%)	53.2 (20.0 - 186.4)	74.7 (24.5 - 194.0)	68.2 (4.8 - 215.2)	0.7

PRISM III - Pediatric Risk for Mortality; MV - mechanical ventilation; HOMA - homeostasis model assessment. The results are expressed as median (range) or number (%).

Blood glucose levels were significantly positively correlated with the PRISM III score and the number of system failures (r = 0.302, p = 0.019, and r = 0.296, p = 0.022, respectively). Blood glucose levels were significantly negatively correlated with the age of patients (r = −0.305), p = 0.006, but there were no statistically significant correlations between BG levels and length of stay or mechanical ventilation duration (p = 0.243 and 0.919, respectively).

We found a significant correlation between BG levels and insulin levels (r = 0.275 and p = 0.013).

Insulin levels were significantly negatively correlated with the number of organ dysfunctions (r = −0.33, p = 0.01), but there were no significant correlations between insulin levels and PRISM III score, length of stay, or mechanical ventilation duration (p = 0.167, 0.382, and 0.435, respectively).

Using HOMA to investigate the pathogenesis of hyperglycemia in critically ill patients (β-cell dysfunction and/or insulin resistance) revealed that 26 (43.3%) critically ill children had low β-cell function (HOMA-%B < 50%), and 18 (30%) critically ill children had low insulin sensitivity (HOMA-%S < 50%). Two children had both β-cell dysfunction and insulin resistance (3.3%).

We found a significant association between low β-cell function and the presence of multi-organ dysfunction, respiratory dysfunction, cardiovascular dysfunction, hematological dysfunction, and the presence of sepsis (Table 3).

**Table 3:** Relationships of β-cell function with study variables

Variables	β-cell function (HOMA-B%)		
	Range	Median	p value
Multi-organ dysfunction			
Yes	5.0 - 240.0	34.5	0.045
No	14.0 - 227.9	62.7	
Respiratory dysfunction			
Yes	5.0 - 240.0	37.0	0.046
No	15.6 - 227.9	62.7	
Cardiovascular dysfunction			
Yes	5.0 - 152.6	26.2	0.009
No	8.3 - 240.0	61.6	
Neurological dysfunction			
Yes	5.0 - 240.0	38.8	0.3
No	14.0 - 227.9	50.3	
Renal dysfunction			
Yes	125.0 - 125.0	125.0	0.3
No	5.0 - 240.0	46.0	
Hepatic dysfunction			
Yes	5.0 - 66.7	21.8	0.2
No	5.5 - 240.0	50.3	
Metabolic dysfunction			
Yes	5.0 - 227.9	36.5	0.3
No	5.5 - 240.0	61.6	
Hematology dysfunction			
Yes	5.0 - 125.0	12.8	0.01
No	5.5 - 240.0	55.9	
Need for MV			
Yes	5.0 - 240.0	42.2	0.5
No	11.8 - 227.9	51.1	
Sepsis			
Yes	5.0 - 174.8	30.3	0.03
No	5.5 - 240.0	62.7	
Glucose level (mg/dL)			
Normal	31.1 - 380.0	124.5	<0.001
126 - 179	15.5 - 125.0	37.0	
≥ 180	5.0 - 72.8	12.9	
Mortality			
Non-survivors	5.0 - 240.0	15.6	0.1
Survivors	8.3 - 227.9	52.4	

HOMA - homeostasis model assessment; MV - mechanical ventilation.

We found that β-cell function was significantly positively correlated with the age of the critically ill children and the number of organ dysfunctions (r = 0.285, p = 0.031 and r = −0.468, p = 0.001, respectively), but it was not significantly correlated with the length of pediatric ICU stay, PRISM III score, or the duration of mechanical ventilation (p = 0.483, 0.057, and 0.795, respectively). Along the same lines, we found a statistically significant positive correlation between insulin sensitivity and the number of system failures (r = 0.298, p = 0.047); however, there were no significant correlations between insulin sensitivity and age of the critically ill children, length of pediatric ICU stay, PRISM score, or duration of mechanical ventilation (p = 0.666, 0.827, 0.913, and 0.515, respectively).

## DISCUSSION

In the current study, hyperglycemia was found in 70% of children receiving care in the pediatric ICU. This finding is in accordance with previous studies, in which a high incidence was associated with a lower cutoff (> 126mg/dL)^(12,39,40)^ and a lower incidence was associated with a higher cutoff (> 150mg/dL).^(1,2,6,34)^

In a comparison of our results with those of other studies using HOMA to classify the pathogenesis of hyperglycemia, our results showed a predominance of isolated β-cell dysfunction in hyperglycemic critically ill children, as evidenced by low HOMA-%B (< 50%) in 26 (43.3%) critically ill children. Consistent with our study, Hacıhamdioğlu et al.^(33)^ also found a predominance of β-cell dysfunction in hyperglycemic critically ill children. In contrast, Verhoeven et al. and Ballestero et al.^(1,34)^ found a predominance of insulin resistance (62% and 50%, respectively), while β-cell dysfunction had a lower incidence (17% and 4%, respectively). Additionally, those studies demonstrated a higher incidence of combined insulin and β-cell dysfunction (21% and 50%, respectively) in comparison with our study (3.3%). These contrasts could be attributed to different cohorts, since Ballestero et al.^(34)^ chose patients with severe hyperglycemia for their cohort and Verhoeven et al.^(1)^ chose patients with meningococcal septicemia.

We found that hyperglycemia (BG > 180mg/dL) was significantly associated with mortality, in accordance with several other studies.^(3,5,7-9,12,39,41)^ Moreover, BG ≥ 180mg/dL was significantly associated with higher PRISM III scores (p = 0.001), and BG level was significantly positively correlated with PRISM III score (r = 0.302, p = 0.019). These findings agree with those of Ballestero et al.,^(34)^ but they differ from the report by Patki and Chougule,^(40)^ in which the PRISM score fell just short of statistical significance. In contrast to our results, Bhutia et al.^(39)^ did not find any association between the severity of illness at admission and the occurrence of hyperglycemia. The difference could be explained by the use of a different score (PIM2) in measuring the severity of illness, different cutoffs, or different cohorts.

We found a significant correlation between multi-organ dysfunction and BG ≥ 180mg/dL. Furthermore, BG level was positively correlated with the number of organ dysfunctions (r = 0.296, p = 0.022, respectively), in agreement with previous studies that evaluated the association of organ dysfunctions with hyperglycemia in critically ill children.^(9,34,39)^ These findings highlight the association of severe clinical conditions with high glucose levels pointing to the severity of underlying illness.

In the present study, respiratory, hematological, and hepatic dysfunctions were more frequent in critically ill children with BG ≥ 180mg/dL. Valerio et al. found that stress hyperglycemia in children was significantly associated with febrile seizures and traumatic injuries.^(42)^

β-cell dysfunction was prominent in respiratory, cardiovascular, and hematological dysfunctions. In a study by Preissig and Rigby,^(5)^ primary β-cell dysfunction, as defined by low endogenous C-peptide production, appeared to be prevalent in critically ill children who had both respiratory and cardiovascular failure, whereas elevated insulin resistance appeared to be the prominent cause of hyperglycemia in children with respiratory failure alone. The difference between the 2 studies may be due to the different analysis of organ dysfunction in our study or to the small subgroup analysis of Preissig and Rigby's study.^(5)^

We did not find a significant correlation between the BG level and the length of stay in the pediatric ICU. Branco and Tasker^(43)^ reported the same finding, but it is in contrast to studies by Bhutia et al.^(39)^ and Patki and Chougule,^(40)^ in which patients with BG ≥ 180mg/dL had a significantly prolonged stay compared with patients with stress hyperglycemia but lower BG levels. This finding denotes that patients with severe hyperglycemia tend to have prolonged pediatric ICU stays for their severe underlying critical illness.

We also demonstrated an association between severe hyperglycemia and severe sepsis and septic shock, which is consistent with Verhoeven et al.,^(1)^ who found that children with meningococcal sepsis and septic shock often develop hyperglycemia.

Insulin levels were significantly negatively correlated with the number of system failures (r = -0.33, p = 0.01), but there were no significant correlations between insulin levels and PRISM III score, length of stay, or mechanical ventilation duration. In contrast to our findings, Preissig and Rigby^(5)^ found that low levels of insulin in critically ill children with hyperglycemia were associated with the severity of illness, increased duration of mechanical ventilation, and a longer pediatric ICU stay.

The differences between our results and those of other studies are due to different study cohorts, but all study conclusions are along the same lines.

One of the limitations of our study was that we did not observe the glucose trends during the hospital stay. Another limitation was the small study cohort.

## CONCLUSIONS

Hyperglycemia was common among critically ill children in our study. Patients with blood glucose ≥ 180mg/dL had a poor clinical outcome, and these levels were associated with a more severe clinical condition and a higher sepsis grade. We identified a higher incidence of low β-cell function as an etiological cause of hyperglycemia in the studied cohort.

We recommend conducting further studies that include a larger number of patients with stress hyperglycemia, and we recommend further evaluation of the homeostasis model assessment to establish its diagnostic ability.
